# Grading by AI makes me feel fairer? How different evaluators affect college students’ perception of fairness

**DOI:** 10.3389/fpsyg.2024.1221177

**Published:** 2024-02-02

**Authors:** Fangyuan Chai, Jiajia Ma, Yi Wang, Jun Zhu, Tingting Han

**Affiliations:** ^1^Graduate School of Education, Beijing Foreign Studies University, Beijing, China; ^2^School of Marxism, Hubei University of Economics, Wuhan, Hubei, China

**Keywords:** higher education evaluation, AI algorithm, fairness perception, information transparency, explanation

## Abstract

**Introduction:**

In the field of education, new technologies have enhanced the objectivity and scientificity of educational evaluation. However, concerns have been raised about the fairness of evaluators, such as artificial intelligence (AI) algorithms. This study aimed to assess college students’ perceptions of fairness in educational evaluation scenarios through three studies using experimental vignettes.

**Methods:**

Three studies were conducted involving 172 participants in Study 1, 149 in Study 2, and 145 in Study 3. Different evaluation contexts were used in each study to assess the influence of evaluators on students’ perception of fairness. Information transparency and explanations for evaluation outcomes were also examined as potential moderators.

**Results:**

Study 1 found that different evaluators could significantly influence the perception of fairness under three evaluation contexts. Students perceived AI algorithms as fairer evaluators than teachers. Study 2 revealed that information transparency was a mediator, indicating that students perceived higher fairness with AI algorithms due to increased transparency compared with teachers. Study 3 revealed that the explanation of evaluation outcomes moderated the effect of evaluator on students’ perception of fairness. Specifically, when provided with explanations for evaluation results, the effect of evaluator on students’ perception of fairness was lessened.

**Discussion:**

This study emphasizes the importance of information transparency and comprehensive explanations in the evaluation process, which is more crucial than solely focusing on the type of evaluators. It also draws attention to potential risks like algorithmic hegemony and advocates for ethical considerations, including privacy regulations, in integrating new technologies into educational evaluation systems. Overall, this study provides valuable theoretical insights and practical guidance for conducting fairer educational evaluations in the era of new technologies.

## Introduction

Imagine at the end of a public elective course you are taking, the teacher grades each student with a final score consisting of everyone’s daily class performance and a final exam. However, you are discontented with your score and have some questions. Is the score given by the teacher fair? Is the marking process transparent? Why did I get this score? Is there an explanation? On second thoughts, if it is not the teacher who is doing the grading, but the artificial intelligence (AI) evaluation system, which could record students’ class behaviors with the help of intelligent technologies such as face and body movement recognition, evaluates your class performance and forms your usual performance using the algorithm logic under set rules, would you think the evaluation process and the grades you get are fairer than the teacher’s evaluation?

In education, the fairness of educational evaluation has always been widely concerned. In the field of higher education, a variety of educational evaluations guides the development direction of colleges and universities and students like a baton. Therefore, the fairness of evaluation will have a direct bearing on the quality of student training. Moreover, with the rapid and iterative development of big data and artificial intelligence technologies, the digital transformation of education has become the future direction. The UN Transforming Education Summit United Nations (2022) pointed out that global education faced serious challenges so education transformation is urgently needed and the power of digital teaching and learning must be fully harnessed. In addition, the Ministry of Education of The people’s Republic of China (2023) strongly called for the international community to promote the digital transformation of education as well.

Thus, AI algorithms has been gradually applied in the field of educational evaluation, including the evaluation of students’ ability and knowledge level, personality and mental health, and so on. For instance, researchers have utilized natural language processing technology to develop models for detecting syntax errors and recognizing argumentation structures, thereby achieving automated and accurate composition scoring (Ruiji et al., 2018). Besides, machine learning techniques are also applied to design an automatic classroom assessment scoring system for the classroom environment, which could observe and record teacher-student interactions while generating instant feedback (James et al., 2018). Hence, there is no doubt that the application of new technologies, such as AI algorithms, has provided new ideas and solutions for educational evaluation reform. Nevertheless, as the traditional evaluators of educational evaluation have always been teachers or educational institutions, students have become familiar with these subjective evaluation habits. Given it, it remains to be tested whether students can feel that the process and the scores are fairer when the evaluator is replaced by an artificial intelligence system. The research claimed that since it was still humans who set the rules for algorithmic evaluation, the subjective bias of the evaluator might still be hidden in the algorithm (Yang, 2022). In other words, the opaque design, unexplainable process, and irrefutable results of AI algorithm evaluations are likely to raise questions about fairness. As a technological tool, it remains controversial as to whether it will enhance or undermine fairness in evaluation. In addition, little research exists on the topic of whether AI algorithmic evaluations are fairer than teacher evaluations, and empirical studies on the influencing mechanism and functional boundary behind it are also lacking.

### Perception of fairness in the field of organizations and education

Fairness is a goal that human society strives for diligently. The fairness perception, as an individual’s subjective feeling and evaluation of justice, is an important indicator of the degree of fairness (Lv and Liu, 2009). The study of the perception of fairness began with Adams’ equity theory, which pointed out that employees tended to care not only about their absolute compensation but also about their relative compensation compared with others, i.e., perception of distributive justice (Adams, 1965). Leventhal et al. (1980) further argued from the perspective of the allocation process that the perception of fairness was closely related to the procedural operations that determined results, such as the development, implementation, and improvement of the allocation system, i.e., procedural justice. Based on previous theories, Bies and Moag (1986) focused on the influence of interpersonal interaction on the perception of fairness during the implementation and proposed the concept of “interactional justice.”

Later, Greenberg and Cropanzano (1993) proposed a taxonomy that reclassified organizational justice into four categories by crosscutting two independent dimensions: (a) category of justice, namely procedural and distributive justice, and (b) focal determinants, including structural and social factors. The four categories are systemic justice, configure justice, informational justice, and interpersonal justice. This categorization further divides interactive justice of Bies and Moag (1986) into informational and interpersonal justice, with information justice being distinct from other types of justice according to Greenberg. Information justice refers to the provision of relevant information and explanations by leaders to their subordinates during the decision-making process, the timely sharing of information, the addressing of concerns related to relevant issues, and the promotion of open and transparent information dissemination (Greenberg and Cropanzano, 1993). Information justice is guided by two important principles or criteria: justification and truthfulness. Managers should provide sufficient justifications for their decision-making actions, and these explanations should be honest, open, and candid (Greenberg and Cropanzano, 1993; Bies, 2005). Building upon this foundation, Colquitt (2001) conducted a series of studies that endorsed the distinction between informational and interpersonal fairness and formalized two approaches for measuring fairness: the direct method and the indirect method. These two approaches correspond to the measurement of subjective and objective aspects of fairness, respectively. The former captures people’s feelings and perceptions of the fairness of an event, while the latter focuses on the inherent fairness of the event itself. This theoretical framework lays the groundwork for measuring perceptions of fairness. By considering the dimensions of justice and integrating the concepts of informational and interpersonal justice, researchers can effectively assess how individuals perceive fairness in various contexts and enhance our understanding of the multifaceted nature of fairness perception.

In the realm of higher education, colleges, and universities can be seen as microcosms of society, while individual classes in which students are enrolled can be likened to organizations. Within the context of educational evaluation, the relationship between the evaluator (whether it is a teacher or an AI algorithm) and the evaluate (the student) can also be compared to the relationship between supervisors and employees. Just as employees place great importance on the fairness of their performance evaluations, students are equally concerned about the fairness with which their academic performance is assessed. Apart from the extensively discussed concepts of distributive and procedural fairness, the integration of AI algorithms into educational evaluation has also stirred concerns among students regarding information fairness. This dimension encompasses issues related to transparent communication, access to information, and the fairness of decision-making processes. Students want to ensure that their performance assessments are conducted in a manner where information is adequately disclosed, unbiased, and consistent. Educational institutions need to address these concerns and create evaluation systems that not only adhere to principles of distributive and procedural fairness but also prioritize information fairness. By promoting transparency, ensuring equal access to information, and implementing fair decision-making practices, colleges and universities can foster an environment where students perceive their evaluations as fair and reliable.

Consequently, the appraisal processes undertaken for college students within the realm of higher education bear a resemblance to performance assessments observed in organizational behavior studies. This parallel provides a rationale for the application of organizational justice theory to the higher education context. Moreover, it is posited that information fairness plays a pivotal role in shaping college students’ trust and perceptions of fairness (Sun and Huang, 2004). In essence, a comprehensive understanding of the fundamental tenets of information fairness theory as it pertains to higher education evaluation is instrumental in elucidating the determinants that sculpt college students’ fairness perceptions during these evaluative interactions.

### The impact of AI algorithm on the perception of fairness in evaluation settings

Nowadays, the AI algorithm has been gradually applied to educational evaluation. In terms of theoretical research, some scholars emphasized that AI algorithm evaluation could achieve innovation in the content and methods of evaluation practice, which was conducive to promoting the development of educational evaluation theory in the context of digital intelligence (Mao et al., 2020; Yigitcanlar et al., 2020; Xu et al., 2022). In terms of empirical research, other scholars have analyzed the specific applications of AI algorithms in student learning behavior evaluation, classroom teaching quality evaluation, higher education enrollment, and so on. For example, AI algorithm models could be used to explain the major issues affecting learners’ learning, predict the trend of changes in students’ learning behavior, assess their learning behavior and status. Afterwards, students can receive effective adaptive feedback and interventions, and the quality of teaching can be improved (Martins and Von Wangenheim, 2022; Xiao et al., 2022).

Adams (1965) emphasized the subjective psychological perception of fairness in his theory of organizational fairness. Afterward, based on this theory, some scholars considered the perception of fairness in performance appraisal as the employees’ overall perception of the fairness of the performance appraisal system in question within the appraisal cycle (Liu et al., 2003; Gu et al., 2020; Ryu and Hong, 2020). In addition, researchers also believed that the perception of fairness refers to an individual’s judgment and emotional experience of the fairness or unfairness of a phenomenon (Lv, 2010; Barsky et al., 2011; Rupp, 2011; Engelmann and Tomasello, 2019). Thus, in the present study, we define “perception of fairness” as an individual’s subjective psychological perception of the fairness or unfairness of the behaviors under specific circumstances.

In terms of the impact of AI algorithms on the perception of fairness, existing research on whether the impacts are positive is disputed. For one thing, given the technical manipulations, the uninterpretability of the AI algorithm as an evaluator was pointed out as a risk (e.g., Adadi and Berrada, 2018; Leslie, 2019; Wang and Wang, 2020). The interpretability of an AI algorithm refers to its ability to be understood and explained, and it is claimed that its operational mechanism, process, and result can be clearly understood by users (Lundberg et al., 2020; Shin, 2021; Cong et al., 2022; Sun, 2022). However, for most users, the complex internal operations of AI algorithm are not easy to understand so there is a “black box” problem for it as an evaluator (Adadi and Berrada, 2018; Guidotti et al., 2018; Rudin, 2019; Luo et al., 2021; Zednik and Boelsen, 2022). As a result, compared to the human evaluation, which can be observed and understood more easily, the unobservable and unintelligible characteristics of AI algorithm evaluation were more likely to raise doubts about the fairness of the evaluation (Burrell, 2016). For another, some scholars argued that those “laymen” who do not know much about algorithms are more likely to have an attitude of “algorithm appreciation.” and believed that AI algorithms had more positive characteristics (Logg et al., 2019; Araujo et al., 2020). That is to say, compared to human evaluation, the AI algorithm, as the evaluator, can collect, process and analyze all kinds of information and data with the help of technologies, and present the development and changes of students in thought, mentality, and behavior in a more scientific and fuller way, bringing a higher perception of fairness to the evaluation object (Wu and Chen, 2014; Williamson, 2017). In addition, the data information obtained in this way is more authentic and reliable, which can enrich the data sources and improve the objectivity of the evaluation (Zhang et al., 2022). In other words, the AI algorithm can eliminate the personal subjective bias of educational evaluators under the logic of digital technology, improving the evaluation method and process and enabling evaluators to obtain a relatively higher perception of fairness.

### Information transparency and the perception of fairness

According to the theory of informational justice, it is necessary to provide employees with relevant information timely based on the implementation of decisions (Greenberg and Cropanzano, 1993; Cropanzano and Greenberg, 1997). Only when the information is sufficiently transparent will employees demonstrate a high degree of trust in the team and thus generate a perception of fairness (Lind, 2001; Zheng and Liu, 2016). In the domain of higher education, transparency of the information is one of the key elements of modern university governance and decision-making, which guarantees students’ rights to information, participation, and supervision, and improves their enthusiasm to participate in university affairs (Damme, 2001; Paloma Sánchez and Elena, 2006; Jihui and Zhilin, 2015; Jongbloed et al., 2018). Hence, information transparency is more than important for students to form a perception of fairness.

Despite that the concept of “transparency” has multiple meanings, the one more widely used is from the perspective of information itself, which defines transparency as a state of information sharing. It is the degree of information disclosure and public provision to external personnel, emphasizing the visibility and availability of information, i.e., the extent to which information is readily accessible, available, and applicable to those who need it or to all stakeholders (Florini, 2007; Tielenburg, 2018; Wang and Ren, 2022). Therefore, in conjunction with informational justification, this study defines “information transparency” in the evaluation as the extent to which valid information, such as the evaluation criteria, methods, and results, is publicly presented and provided to evaluation objects.

In organizational studies on how information transparency affects the perception of fairness, it has been pointed out that differences in information fairness or low information transparency constructed information barriers that subsequently create suspicion and mistrust among these members (Carless, 2009; He et al., 2017). In other words, the level of presentation of information related to evaluation criteria, methods, and results by evaluators is one of the key factors affecting the evaluation objects’ perception of fairness (Sweeney and McFarlin, 1993; Dochy et al., 1999; Van Berkel et al., 2021). Therefore, information transparency is a vital link to promote the formation of the perception of fairness (Prahalad and Ramaswamy, 2002; Pei et al., 2021). To be more specific, when evaluation criteria are not made public in a way that is easy to understand, the perception of fairness of the person being evaluated will decline (Chan Kim and Mauborgne, 1998; Yuan et al., 2022). For instance, some scholars emphasized that employers had a responsibility to inform employees of the procedures and standards of their performance evaluation system and to disclose the relevant evaluation methods promptly to ensure the organization and evaluation were in a relatively transparent environment, which was a basic condition for employees to develop a perception of fairness (Belogolovsky and Bamberger, 2014; Schnackenberg and Tomlinson, 2016; Wei and Sun, 2022). Moreover, the transparency of the evaluation method is an effective basis for judging whether the evaluation is fair and reasonable. If the methods used by the evaluator are consistent, accurate, unbiased, reasonable, and transparent, then the evaluation object will be able to establish a high perception of fairness (Rasooli et al., 2019). For instance, the evaluation methodology employed by AI algorithms constitutes a comprehensive digital service based on algorithmic rules and programmatic design. In comparison to teacher evaluations, AI algorithmic assessments offer greater clarity and transparency, eliminating personal emotional factors that may bias evaluators and consequently yielding a heightened perception of fairness. To illustrate this, consider the issue of fairness in the university admissions process. When employing AI algorithms for student admissions evaluations, the algorithms assess and compare candidates based on a range of objective criteria and data indicators such as academic performance and standardized test scores. This evaluation approach remains unaffected by subjective biases, thereby avoiding deviations driven by evaluators’ personal preferences or emotions (e.g., Dennis, 2018; Marcinkowski et al., 2020; Ahmad et al., 2022). In contrast, teacher evaluations may be susceptible to subjective influences, such as individual preferences or emotional factors, which can potentially undermine fairness. Moreover, the utilization of AI algorithms in assessment processes enhances transparency. This approach affords students a lucid grasp of the evaluative criteria, methodologies, and their application to individual scenarios. For instance, certain academics have developed a framework for certifying learning outcomes grounded in blockchain technology, leveraging its inherent characteristics of public verifiability, authenticity of content, and resistance to alteration, thereby ensuring a true and precise documentation of learners’ educational trajectories (Wu et al., 2018). Evaluators can scientifically validate learners’ accomplishments by examining the recorded data, thereby furnishing a transparent, equitable, and objective certification of achievements. This level of clarity enables students to more thoroughly understand the basis of their assessments and facilitates the pursuit of clarification or justification of the results. In juxtaposition, traditional instructor-led evaluations may suffer from a lack of such transparency, potentially leaving students in the dark regarding the exact standards and methodologies utilized in their assessment (Daud, 2022).

In the context of higher education, students’ perception of fairness in evaluations is significantly shaped by the transparency of information provided (e.g., Awad and Krishnan, 2006; Kizilcec, 2016). To elaborate, when students are given comprehensive insights into the criteria and methodologies used in the evaluation process, it fosters a stronger sense of trust toward the evaluator. This, in turn, enhances their perception of fairness. However, if the evaluation process is veiled in ambiguity and lacks transparency, it leaves students grappling with uncertainty (Baartman et al., 2007; Lebovitz et al., 2022). This lack of clarity inevitably leads to a sense of perceived unfairness, especially when the results do not align with their expectations.

### Explanation and the perception of fairness

Organizational studies have shown that in addition to the basic information about the criteria, whether the supervisor justified the decision influenced the employee’s perception of information fairness as well (Bies and Shapiro, 1987; Dulebohn and Ferris, 1999; Hollensbe et al., 2008; Hewett and Leroy, 2019). If supervisors communicated with their employees promptly and provided them with specific reasons for the decision, employees not only perceived their leaders as sincere, transparent, and trustworthy, but also had positive psychological and emotional experiences, such as the perception of fairness (Chan Kim and Mauborgne, 1998; Gardner et al., 2005). In short, providing an explanation of the procedure or result is beneficial to strengthen trust and bring a higher perception of fairness to the evaluator (Shulner-Tal et al., 2022).

At present, the concept of “explanation” is not yet universally agreed upon academically. Some scholars believed that explanation was the logical and meaningful information provided by the decision maker about the result, which could explain the relationship between the input contents and output results (Goodman and Flaxman, 2017). It has also been claimed that explanation was the process of providing a multidimensional in-depth analysis and interpretation of the actual results, reasonably explaining the causes of the changes, the connections between things and the rules by which things develop, and making guiding suggestions (Wang, 2016). Thus, in essence, explanation is a kind of information transfer, whose basic elements include the interpreter, the receiver, and the explanatory information, and whose content often involves the comparison of target levels and the analysis of actual results. In summary, this study defines “explanation” as the process of delivering the evaluation result to the evaluation object in some form after comparing their current performance with the expected standard and explaining the specific reasons for that result to them.

Most of the current research on explanation and the perception of fairness focused on organizational behaviors. Research in this area has found a strong link between the process of communicating performance evaluation results to the employees involved and their perception of fairness (e.g., Niehoff and Moorman, 1993; Kluger and DeNisi, 1996; Lemons and Jones, 2001). Specifically, performance evaluations are of great importance to employees as they directly impact their interests. Not only do the evaluation results influence employees’ perception of distributive fairness, but the communication, opportunities for appeals, and explanations regarding the evaluation outcomes also play a significant role in shaping their perception of procedural fairness (Magner et al., 1994; Fatt et al., 2010; Sudin, 2011; Alterman et al., 2021). When employees receive their manager’s evaluation of their past work without understanding the rationale behind the results or the appropriate improvement measures based on that evaluation, they may inevitably question the fairness of the assessment (Gupta and Kumar, 2012; Gu et al., 2020). Therefore, if performance evaluations are not thoroughly explained and feedback is not provided to employees, it can lead to a certain level of job dissatisfaction and foster a perception of organizational unfairness. By contrast, adequate and effective explanations can make the overall performance evaluation fairer and increase the perception of fairness among employees, motivating them to work passionately and develop their capabilities (Xiong, 2011; De Clercq and Pereira, 2020). Similar to performance evaluation, educational evaluation is also a management tool to improve the quality of education. Evaluating and explaining the behavior of college students in education activities can not only diagnose the “shortcomings” and deficiencies in their studies but also provide important quality standards and directions for their future development.

Therefore, whether or not an explanation is given after the evaluation, and whether or not college students understand the reasons for this evaluation, will also affect their perception of fairness in educational evaluation. If these students can only see the evaluation results but do not have any access to an explanation of the results, then when there is a gap between the evaluation results and their psychological expectations, they will inevitably question the rationality of the evaluation results and the fairness of the evaluation process. Otherwise, if they are provided with accurate and comprehensive explanatory information, they will tend to feel fairer about the results, regardless of whether the evaluator is an AI or a teacher.

This study aims to investigate the impact of evaluator type (teachers vs. AI algorithms) on students’ perceptions of fairness in higher education evaluation, as well as the role of information transparency and interpretation in this process. The research questions are: Do college students perceive a higher level of fairness when an AI algorithm is used as the evaluator compared to traditional teacher evaluation? How does the use of an AI algorithm affect the fairness perception of college students? What are the boundary conditions for this influence on fairness perception?

**Figure 1:**
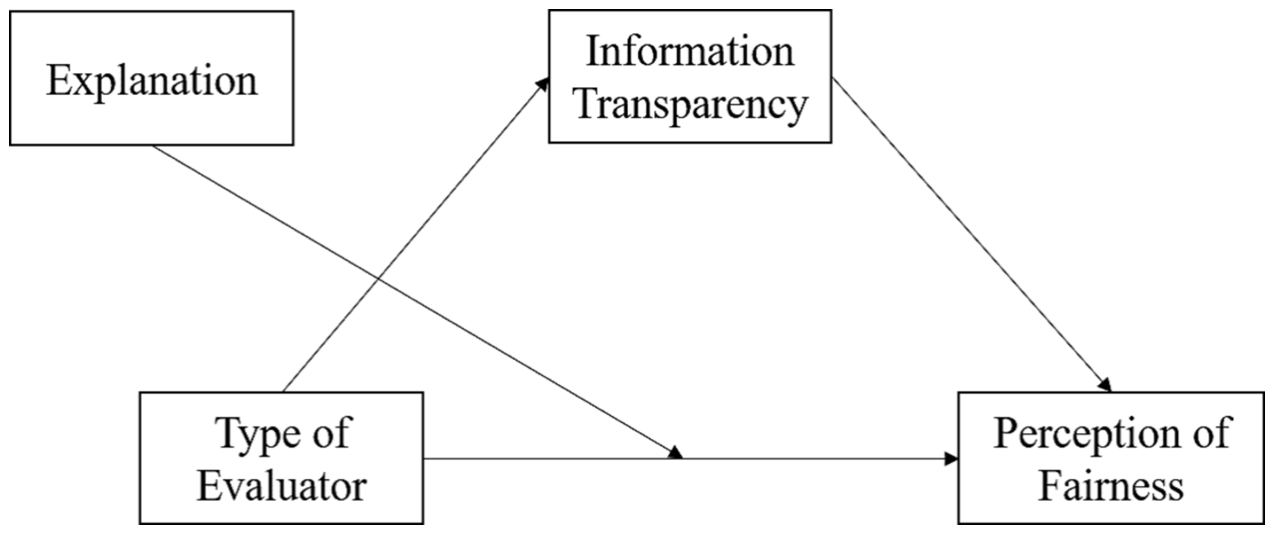
Hypothetical model of the role of information transparency, explanation in the relationship between the type of evaluator and the perception of fairness.

The hypotheses of the study are: (a) College students attribute higher perceptions of fairness to AI algorithms than to teachers as evaluators; (b) Information transparency serves as a mediating variable between different evaluators and the perception of fairness, such that college students perceive greater information transparency from an AI algorithm and therefore have higher perceptions of fairness; (c) The explanation of evaluation results moderates the influence of evaluators on fairness perceptions, meaning that when the reasons for evaluation decisions are explained to college students, the influence of evaluators on fairness perceptions diminishes. Based on these hypotheses, a hypothetical model of the role of information transparency and explanation in the relationship between the type of evaluator and the perception of fairness is proposed in this study (see Figure 1).

## Study 1: the impact of different evaluators on college students’ perception of fairness

Based on three types of educational evaluation contexts (diagnostic, formative, and summative evaluations), Study 1 intends to investigate the effects of different evaluators (AI algorithms vs. teachers) on college students’ perception of fairness and whether the effects differ.

### Methods

#### Participants

The participants were randomly recruited on the online platform Credamo, and 7 were excluded for completing the questionnaire in too short a time or not meeting the response requirements. All participants must be current Chinese undergraduate students. We excluded individuals who had previously participated in similar decision-making or cognitive psychology studies to avoid any potential confounding effects of prior exposure to similar experimental paradigms. Specific demographic information of participants is as follows (see Table 1). The final sample included 172 participants (*M_age_* = 21.22, *SD_age_* = 1.57), of which 71 were male and 101 were female. Freshmen, sophomores, juniors, and seniors represented 8.2, 23.8, 39.6, and 28.4% of the sample, respectively. The participants were selected from a diverse range of majors, encompassing 10 different categories such as literature, engineering, law, medicine, and others. Among the subjects, 31.4% hailed from China’s double-first-class universities, while the remaining 68.6% were enrolled in ordinary universities.

**Table 1:** Demographic information of participants in Study 1.

Demographic characteristic		Frequency	Percentage	Demographic characteristic		Frequency	Percentage
Gender	Female	101	58.7%	Major	Law	8	4.8%
	Male	71	41.3%		Engineering	48	27.9%
Age	18	6	3.5%		Management	28	16.3%
	19	15	8.7%		Education	12	7.0%
	20	32	18.6%		Economics	17	9.9%
	21	54	31.4%		Science	16	9.3%
	22	39	22.7%		Literature	22	12.8%
	23	12	7.0%		Medicine	12	7.0%
	24	4	2.3%		Arts	6	3.5%
	25	10	5.8%		Philosophy	3	1.7%
Grade	Freshmen	41	8.2%	University type	Double-first-class	54	31.4%
	Sophomore	68	23.8%				
	Junior	49	39.6%		Ordinary	118	68.6%
	Senior	14	28.4%				

#### Design

The current study employed the experimental vignette methodology, more specifically the paradigm of *paper people study*. This method involved presenting participants with meticulously designed and realistic scenarios related to educational evaluation, aiming to assess their perception of fairness. By employing this methodology, the study successfully enhanced the authenticity of the experiment while effectively manipulating and controlling independent variables, such as the evaluators in this study. Additionally, the experimental vignette methodology has demonstrated its significant value in investigating causal relationships between variables within sensitive contexts (Aguinis and Bradley, 2014). It has gained considerable recognition and widespread application across diverse academic disciplines, particularly finding popularity in the realm of ethical decision-making (e.g., Finch, 1987; Hyman and Steiner, 1996; Clifford et al., 2015; Ekici et al., 2023). The study adopted a single-factor within-subject design. The independent variable was the evaluator (AI algorithm vs. teacher), and the dependent variable was students’ self-reported perception of fairness for different evaluators. This design scrutinizes the variations in participants’ perceptions of fairness among different evaluators for the same participant, thereby further quantifying the influence of the evaluator on students’ perceptions of fairness.

#### Materials and variable measurement

##### Descriptive situational materials

Referring to Bloom’s classic classification of educational evaluation, this study classified evaluation into diagnostic evaluation, formative evaluation, and summative evaluation (Bloom, 1969, 1987), based on the timing and role of its implementation in teaching and learning. By these types mentioned above, students’ learning starting point (current abilities), learning process and learning outcomes could be evaluated, respectively. Taking into account the reality of educational evaluation in China, descriptive situational materials were developed around each of the three types of evaluation. Two scenarios were created for each type of assessment to facilitate the selection of the most appropriate one through expert evaluation. The diagnostic evaluation comprised two contexts: students’ oral proficiency test at the beginning of the school year and a pre-class quiz. The formative evaluation included a midterm examination and the assessment of regular performance. Lastly, the summative evaluation encompassed the evaluation of the final paper and the assessment of the final presentation. To ensure the quality of the materials, we enlisted the help of 10 master’s degree students majoring in education to review the content and language of the texts. Additionally, three professors specializing in psychology were invited to evaluate all the materials. Finally, three kinds of descriptive situational materials were obtained regarding the oral proficiency test, the scoring of regular grades, and the evaluation of the final paper.

In this study, the independent variable was managed by changing the evaluators in three descriptive situational materials. The first half of each material was the same, e.g., “Before taking a public English class, your university will organize a formal speaking test. The level of your test score will determine whether you are placed in a higher-level class or a lower-level class.” Next, each scenario will include both an AI algorithm evaluation and a teacher evaluation. The AI algorithm evaluation scenario was described as “The university has applied an AI evaluation system and the speaking test is in the form of a human-computer dialog, after which the AI algorithm scores you based on your pronunciation, expression, fluency, etc.” while the teacher evaluation scenario was described as “It’s test is a face-to-face oral test and at the end of the test, the teacher scores you based on your pronunciation, expression, fluency, etc.” The AI algorithm was assigned as 1 and the teacher was assigned as 2.

##### Student’s perception of fairness

There are two types of measuring methods for assessing the perception of fairness: “indirect measurement” and “direct measurement.” The former focuses on evaluating the fairness of the event itself. For instance, in a competition for outstanding students, indirect measures would involve questions such as “Did the evaluation committee treat each applicant equally?,” “Was the committee’s decision based on accurate and sufficient information?.” The latter, on the other hand, places greater emphasis on individuals’ subjective feelings. For instance, in a competition for outstanding students, direct measures would inquire about individuals’ perceptions through questions such as “How fair do you feel you were treated in the selection process?,” and “How fair do you think the committee was in the way it made its decision?” (Colquitt and Shaw, 2013). Given that this study focused more on the subjective perceptions of students as the object of educational evaluation, the direct measurement was adopted and only one item was used, i.e., after reading the material, the participants were asked to answer how fair he or she thought the teacher/AI algorithm was as the evaluator in this context. The perception of fairness was scored on a 7-point scale from 1 to 7, ranging from “very unfair” to “very fair.” The Cronbach’s α coefficient for the scale in this study was 0.67.

#### Procedures

All the participants were asked to read three descriptive situational materials in turn, and intuitively rated the fairness of the AI algorithm as the evaluator and the teacher as the evaluator, respectively. Finally, they filled in the demographic information.

### Results

**Figure 2:**
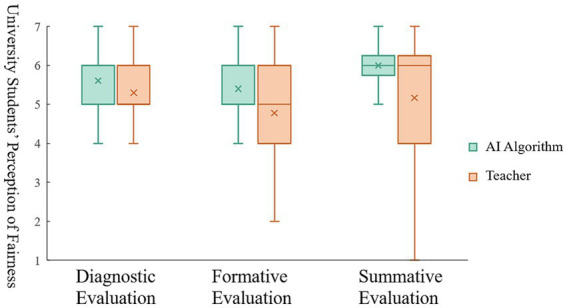
Difference in the perception of fairness between AI algorithm evaluation and teacher evaluation.

With the evaluator as the independent variable and the perception of fairness of the three evaluation types as the dependent variable, a single-factor analysis of variance (ANOVA) was conducted for the samples. The results showed (see Figure 2) that in diagnostic evaluation, participants’ perception of fairness for AI algorithm evaluation was significantly higher than that for teacher evaluations (*M_AI_* = 5.60, *SD* = 1.12, *M_Teacher_* = 5.30, *SD* = 0.96; *F*(1, 342) = 7.24, *p* < 0.01). In formative evaluation, participants’ perception of fairness for AI algorithm evaluation was significantly higher than that for teacher evaluations (*M_AI_* = 5.40, *SD* = 1.21, *M_Teacher_* = 4.78, *SD* = 1.15; *F*(1, 342) = 23.87, *p* < 0.001). In summative evaluation, participants’ perception of fairness for AI algorithm evaluation was significantly higher than that for teacher evaluations as well (*M_AI_* = 5.48, *SD* = 1.17, *M_Teacher_* = 5.20, *SD* = 1.22; *F*(1, 349) = 4.26, *p* < 0.05). Among the three types of evaluation, the effect-size coefficients for the evaluator were *η^2^_diagnostic evaluation_* = 0.03*η^2^_formative evaluation_* = 0.08, *η^2^_summative evaluation_* = 0.02.^1^ These coefficients suggest that AI algorithm evaluation consistently led to higher perceived fairness for students compared to traditional teacher evaluation, regardless of the specific type of evaluation being conducted. This implies that students perceive AI algorithm evaluation as fairer and more unbiased in comparison to evaluations conducted by human teachers, irrespective of whether the evaluation is diagnostic, formative, or summative in nature.

Study 1 verifies the impact of the evaluator on the perception of fairness, i.e., college students’ perception of fairness brought by the AI algorithm as an evaluator is significantly higher than that of teachers. Next, Study 2 and Study 3 will further reveal the mechanism of the evaluator’s influence on the perception of fairness, examine the role of information transparency, and analyze the boundary conditions under which the evaluator’s influence on the perception of fairness may diminish.

## Study 2: the mediating role of information transparency on the influence of different evaluators on college students’ perception of fairness

Study 2 followed the design of Study 1 but additionally included a new variable information transparency. It intended to further examine the mediating role of information transparency and to investigate the main effect of different evaluators (AI algorithm/teacher) on college students’ perception of fairness and the mediating effect of information fairness.

### Methods

#### Participants

The participants were randomly recruited on the online platform Credamo, and 4 were excluded for completing the questionnaire in too short a time or not meeting the response requirements. All participants must be current Chinese undergraduate students. We excluded individuals who had previously participated in similar decision-making or cognitive psychology studies to avoid any potential confounding effects of prior exposure to similar experimental paradigms. Specific demographic information of participants is as follows (see Table 2). The final sample included 149 participants (*M_age_* = 21.29, *SD_age_* = 1.57), of which 58 were male and 91 were female. Freshmen, sophomores, juniors, and seniors constituted 6.0, 23.4, 40.4, and 30.2% of the participants, respectively. The participants represented a diverse range of majors, including literature, engineering, law, medicine, and others, encompassing a total of 10 different categories. Within the sample, 30.2% of the subjects were enrolled in China’s double-first-class universities, while the remaining 69.8% attended ordinary universities.

**Table 2:** Demographic information of participants in Study 2.

Demographic characteristic		Frequency	Percentage	Demographic characteristic		Frequency	Percentage
Gender	Female	91	61.1%	Major	Law	8	5.4%
	Male	58	38.9%		Engineering	41	27.5%
Age	18	5	3.4%		Management	20	13.4%
	19	10	6.7%		Education	12	8.1%
	20	27	18.1%		Economics	14	9.4%
	21	48	32.2%		Science	13	8.7%
	22	36	24.2%		Literature	21	14.1%
	23	10	6.7%		Medicine	11	7.4%
	24	4	2.7%		Arts	6	4.0%
	25	9	6.1%		Philosophy	3	2.0%
Grade	Freshmen	9	6.0%	University type	Double-first-class	54	30.2%
	Sophomore	35	23.5%				
	Junior	60	40.3%		Ordinary	118	69.8%
	Senior	45	30.2%				

#### Design

This study employed the experimental vignette methodology as study 1. The independent variables were the evaluator (AI algorithm vs. teacher) and the information transparency (high transparency vs. low transparency), and the dependent variable was students’ self-reported perception of fairness for different evaluators.

#### Materials and variable measurement

Descriptive situational material and manipulation of evaluators and measurement upon fairness perceptions were kept consistent with Study 1, but the measurement of information transparency was additionally added as a mediator variable. The Information Transparency Scale developed by Liu et al. was selected as the measurement tool and revised to fit the experimental context, resulting in the retention of six items, including two reverse scoring items (Liu et al., 2015). The questions included “The teacher/AI algorithm relies on more useful information,” “The information relied on by the teacher/AI algorithm is easier to obtain,” “The information relied on by the teacher/AI algorithm is not sufficient” etc. The scale was scored on a 7-point scale from 1 to 7, ranging from “strongly disagree” to “strongly agree.” The Cronbach’s *α* coefficient for the scale in this study was 0.80.

#### Procedures

All participants were asked to read three descriptive situational materials in turn, and intuitively rated the fairness of the AI algorithm as the evaluator and the teacher as the evaluator, then completed the scale of information transparency. Finally, they filled in the demographic information.

### Results

#### Descriptive statistics and correlation analysis

The correlation analysis of the main variables of Study 2 was performed and the results are shown in Table 3. The results showed that there was a significant negative correlation between evaluator and information transparency and the perception of fairness of the three types of evaluation (*r* = −0.66, *p* < 0.01; *r* = −0.17, *p* < 0.01; *r* = −0.28, *p* < 0.01; *r* = 0.12, *p* < 0.5), which means that information transparency and the perception of fairness is higher in the AI algorithm evaluation condition. Besides, there was a significant positive correlation between information transparency and the perception of fairness of the three types of evaluation (*r* = 0.36, *p* < 0.01; *r* = 0.42, *p* < 0.01; *r* = 0.37, *p* < 0.01). The above analysis results are in line with the theoretical expectations of this study and provide preliminary support for the research hypotheses.

**Table 3:** Descriptive statistics of variables and correlation coefficient matrix in Study 2.

Variable	*M* ± *SD*	1	2	3	4	5
1. Type of evaluator	1.50 ± 0.50					
2. Information transparency	3.99 ± 1.23	−0.66**				
3. Perception of fairness in diagnostic evaluation	5.46 ± 1.10	−0.17**	0.36**			
4. Perception of fairness in formative evaluation	5.10 ± 1.23	−0.28**	0.42**	0.33**		
5. Perception of fairness in summative evaluation	5.31 ± 1.16	−0.12*	0.37**	0.36**	0.49**	

Coding of evaluator types: AI evaluation = 1, Teacher = 2.

*p < 0.05, **p < 0.01, ***p < 0.001.

#### Mediating effect test

This study used PROCESS developed by Preacher and Hayes to test the mediating effect of information transparency (Preacher and Hayes, 2004). With the evaluator as the independent variable and the perception of fairness of the three types of evaluation as the dependent variable, the general mediating effect was tested, and the results were shown in Table 4.

**Table 4:** Mediating effect test of information transparency.

Type	Equation	Dependent variable	Independent variable	*R* ^2^	*F*	*β*	*SE*	95%CI
Diagnostic evaluation	1	Perception of fairness	Type of evaluator	0.01	8.69**	−0.37**	0.13	[−0.62, −0.12]
	2	Information transparency	Type of evaluator	0.44	227.88***	−1.62***	0.11	[−1.83, −1.41]
	3	Perception of fairness	Information transparency	0.14	24.12***	0.40***	0.06	[0.27, 0.52]
			Type of evaluator			0.27	0.16	[−0.04, 0.58]
Formative evaluation	4	Perception of fairness	Type of evaluator	0.07	24.84***	−0.69***	0.22	[−0.96, −0.41]
	5	Information transparency	Type of evaluator	0.44	227.88***	−1.62***	0.11	[−1.83, −1.41]
	6	Perception of fairness	Information transparency	0.17	30.98***	0.41***	0.07	[0.28, 0.56]
			Type of evaluator			−0.02	0.17	[−0.37, 0.33]
Summative evaluation	7	Perception of fairness	Type of evaluator	0.01	4.26*	−0.28**	0.13	[−0.54, −0.01]
	8	Information transparency	Type of evaluator	0.44	227.88***	−1.62***	0.11	[−1.83, −1.41]
	9	Perception of fairness	Information transparency	0.16	27.53***	0.48***	0.07	[0.34, 0.61]
			Type of evaluator			−0.49**	0.16	[0.17, 0.82]

Coding of evaluator types: AI evaluation = 1, Teacher = 2.

*p < 0.05, **p < 0.01, ***p < 0.001.

**Figure 3:**
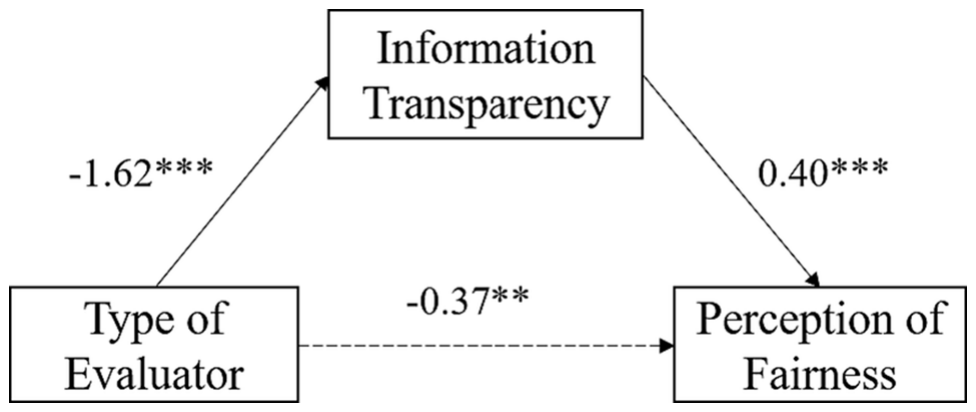
Mediating effect model of information transparency in diagnostic evaluation.

In diagnostic evaluation, equation 1 showed that the evaluator significantly predicted the perception of fairness (*β* = −0.37, *SE* = 0.13, *p* < 0.01). According to equation 2, the evaluator significantly predicted information transparency (*β* = −1.62, *SE* = 0.11, *p* < 0.001). And following equation 3, when both the evaluator and information transparency entered the regression equation, the evaluator could not significantly predict the perception of fairness (*p* > 0.05), whereas information transparency significantly predicted the perception of fairness (*β* = 0.40, *SE* = 0.06, *p* < 0.001). Therefore, the bias-corrected and accelerated bootstrap indicated that information transparency played a full mediating effect between the evaluator and the perception of fairness, with the mediating effect accounting for 63.65% of the total effect (See Figure 3).

**Figure 4:**
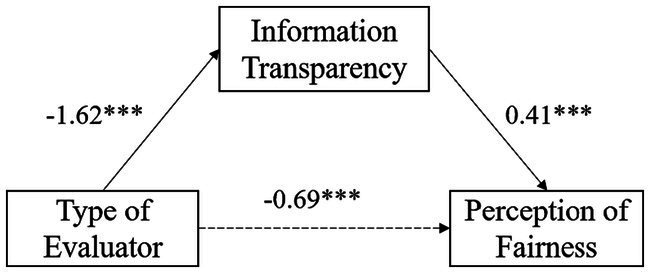
Mediating effect model of information transparency in formative evaluation.

In formative evaluation, equation 1 showed that the evaluator significantly predicted the perception of fairness (*β* = −0.69, *SE* = 0.22, *p* < 0.001). According to equation 2, the evaluator significantly predicted information transparency (*β* = −1.62, *SE* = 0.11, *p* < 0.001). And following equation 3, when both the evaluator and information transparency entered the regression equation, the evaluator could not significantly predict the perception of fairness (*p* > 0.05), whereas information transparency significantly predicted the perception of fairness (*β* = 0.41, *SE* = 0.07, *p* < 0.001). Therefore, information transparency played a full mediating effect between the evaluator and the perception of fairness, with the mediating effect accounting for 49.05% of the total effect (See Figure 4).

**Figure 5:**
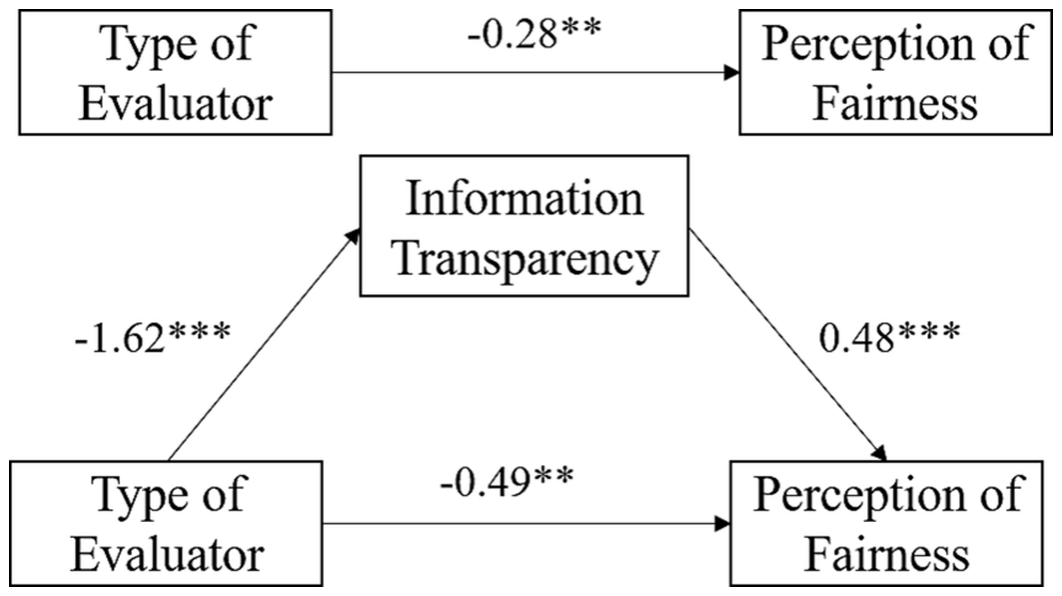
Mediating effect model of information transparency in summative evaluation.

In summative evaluation, equation 1 showed that the evaluator significantly predicted the perception of fairness (*β* = −0.28, *SE* = 0.13, *p* < 0.01). According to equation 2 a, the evaluator significantly predicted information transparency (*β* = −1.62, *SE* = 0.11, *p* < 0.001). And following equation 3, when both the evaluator and information transparency entered the regression equation, the evaluator was still able to significantly predict the perception of fairness (*β* = −0.49, *SE* = 0.16, *p* < 0.01), and information transparency also significantly predicted the perception of fairness (*β* = 0.48, *SE* = 0.07, *p* < 0.001). Therefore, the bias-corrected and accelerated bootstrap indicated that information transparency played a partial mediating effect between the evaluator and the perception of fairness, with the mediating effect accounting for 73.52% of the total effect (See Figure 5).

Hence, in general, college students perceive that the AI algorithm, as the evaluator, has higher information transparency, and thus have higher perceptions of fairness.

## Study 3: the moderating effect of explanation on the influence of different evaluators on college students’ perception of fairness

Study 3 followed the design of Study 1 and 2, intended to further investigate the main effect of different evaluators (AI algorithm vs. teacher) on college students’ perception of fairness and examine the moderating effect of explanation.

### Methods

#### Participants

Taking college students as the basic criteria, the participants were randomly recruited on the online platform Credamo, and 5 were excluded for completing the questionnaire in too short a time or not meeting the response requirements. Specific demographic information of participants is as follows (see Table 5). The final sample included 145 participants (*M_age_* = 21.31, *SD_age_* = 1.67), of which 56 were male and 89 were female. Freshmen, sophomores, juniors, and seniors comprised 5.5, 26.9, 41.4, and 26.2% of the participant pool, respectively. These participants represented a diverse range of majors, including literature, engineering, law, medicine, and others, spanning 10 different categories. Of the subjects, 33.8% were hailed in China’s double-first-class universities, while the remaining 66.2% were from ordinary universities.

**Table 5:** Demographic information of participants in Study 3.

Demographic characteristic		Frequency	Percentage	Demographic characteristic		Frequency	Percentage
Gender	Female	56	38.6%	Major	Law	6	4.1%
	Male	89	61.4%		Engineering	41	28.3%
Age	18	4	2.8%		Management	23	15.9%
	19	12	8.3%		Education	9	6.2%
	20	31	21.4%		Economics	12	8.3%
	21	37	25.5%		Science	26	17.9%
	22	31	21.4%		History	3	2.1%
	23	20	13.8%		Literature	15	10.3%
	24	4	2.8%		Medicine	6	4.1%
	25	4	2.8%		Arts	4	2.8%
	26	2	1.4%				
Grade	Freshmen	8	5.5%	University type	Double-first-class	49	33.8%
	Sophomore	39	26.9%				
	Junior	60	41.4%		Ordinary	96	66.2%
	Senior	38	26.2%				

#### Design

The study adopted a mixed design of 2 (evaluator: AI algorithm/teacher) × 2 (with explanation/without explanation). The independent variable was the evaluator (within-subject design), and the dependent variable was students’ self-reported perception of fairness. More specifically, in this study, each participant was asked to read materials about the AI algorithm and the teacher’s evaluation, but one group received a situation with an explanation while the other group received a situation without an explanation. By comparing the differences between these two groups, the moderating effect of explanation could be examined.

#### Materials and variable measurement

The descriptive situational material and the manipulation of evaluators and measurement employed in Study 1 were maintained for consistency in this study. Additionally, an explanatory variable was introduced into the material. To illustrate with formative evaluation as an example, it is important to note that in China, university courses typically involve two grades. The first grade is the final exam grade, which reflects the comprehensive learning outcomes of the student for the entire semester and can be considered a summative evaluation. The second part is the regular grade, which takes into account classroom performance, coursework, and other factors, and captures the student’s performance during the learning process. The first half of the reading material was the same for both groups: “All optional courses’ scores at your university consist of a regular grade and a final exam grade. The final exams are all objective questions with standard answers; the regular grades are given entirely by the teacher of the course.” “All optional courses’ scores at your university consist of a regular grade and a final exam grade. The final exams are all objective questions with standard answers; the regular grades are given entirely by the AI evaluation system applied by the university.” And then, the materials from the group of participants for whom explanations were provided were “After the course is over, the teacher scores your class performance and provides a note to explain the basis for giving that grade.” and “After the course is over, the AI evaluation system scores your class performance and provides a note to explain the basis for giving that grade.” However, the materials from the group without explanation were “After the course was over, the teacher scores your class performance, but does not provide any explanation of the basis for the grade.” and “After the course was over, the AI evaluation system scores your class performance, but does not provide any explanation of the basis for the grade.”

#### Procedures

The participants in each group were asked to read three descriptive situational materials in turn, and intuitively rated the fairness of the AI algorithm as the evaluator and the teacher as the evaluator, then completed the scale of information transparency. Finally, they filled in the demographic information.

### Results

#### Descriptive statistics and correlation analysis

The correlation analysis of the main variables of Study 3 was performed and the results are shown in Table 6. The results showed that there was a significant negative correlation between the evaluator and the perception of fairness of the three types of evaluation (*r* = −0.18, *p* < 0.01; *r* = −0.14, *p* < 0.01; *r* = −0.10, *p* < 0.5). Besides, there was a significant positive correlation between the explanation and the perception of fairness of the three types of evaluation (*r* = 0.61, *p* < 0.01; *r* = 0.58, *p* < 0.01; *r* = 0.59, *p* < 0.01). The above analysis results are in line with the theoretical expectations of this study that the presence or absence of explanation will influence the evaluators’ perception of fairness.

**Table 6:** Descriptive statistics of variables and correlation coefficient matrix in Study 3.

Variable	*M* ± *SD*	1	2	3	4	5
1. Type of evaluator	1.50 ± 0.50					
2. Explanation	1.50 ± 0.50	0.00				
3. Perception of fairness in diagnostic evaluation	4.94 ± 1.51	−0.18**	0.61**			
4. Perception of fairness in formative evaluation	4.98 ± 1.50	−0.14**	0.58**	0.74**		
5. Perception of fairness in summative evaluation	5.06 ± 1.41	−0.10*	0.59**	0.74**	0.76**	

Coding of evaluator types: AI evaluation = 1, Teacher = 2; coding of explanation: Without explanation = 1, With explanation = 2.

*p < 0.05, **p < 0.01, ***p < 0.001.

#### Test for moderating effect

With the evaluator as the independent variable, the perception of fairness of the three types of evaluation as the dependent variable, and the explanation as the moderator variable, Model 1 in the PROCESS program was adopted to test for moderating effects after all continuous variables were centralized.

**Figure 6:**
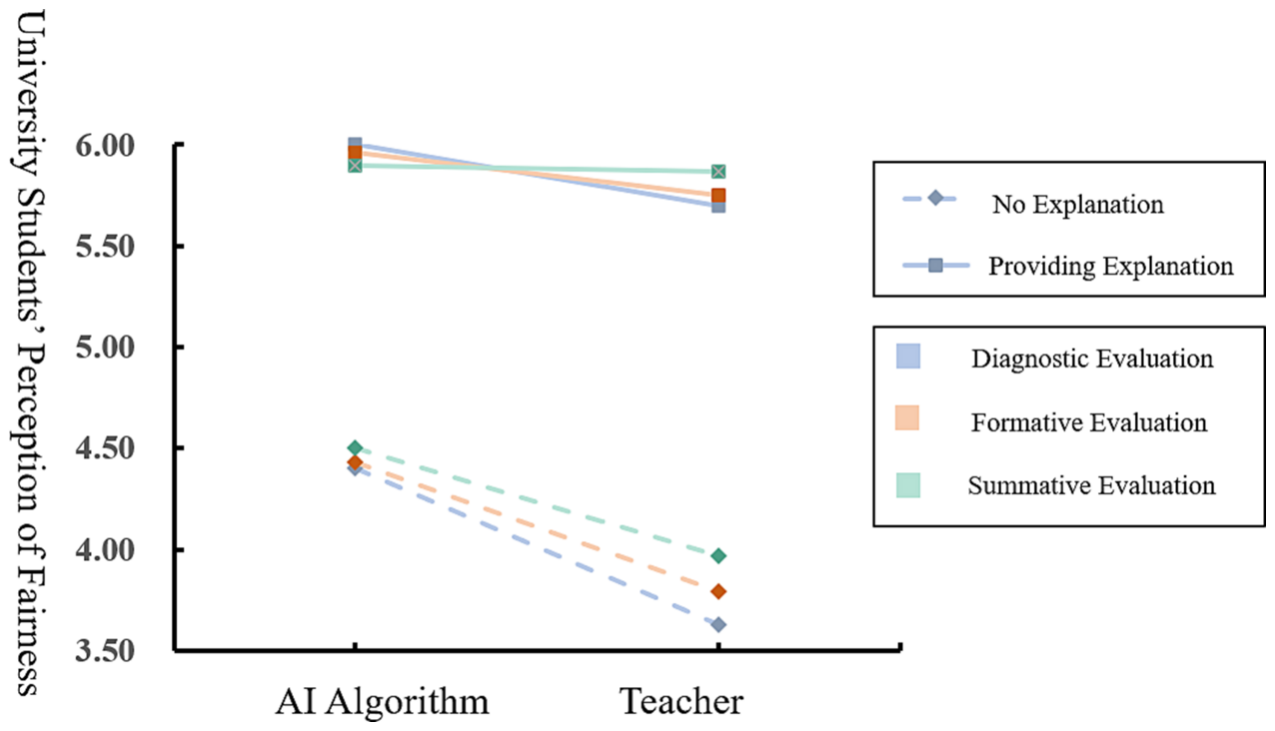
Moderating effect model of explanation.

The results showed that the interaction between the evaluator and the explanation could have a significant positive effect on the perception of fairness in diagnostic evaluation (*β* = 0.43, *SE* = 0.19, *p* < 0.05), and the specific moderating effect was shown in Figure 6. Moreover, according to the results, when there was no explanation, there was a significant difference between the student’s perception of fairness brought by the AI algorithm and that brought by the teacher (*M_AI_* = 4.40, *M_Teacher_* = 3.63, *t*(145) = −5.60*p* < 0.001). However, when there was an explanation, the influence of the evaluator on the perception of fairness diminished, and the difference between the perception of fairness under the two evaluators was no longer significant (*M_AI_* = 6.03, *M_Teacher_* = 5.70, *t*(145) = 2.42, *p*>0.05).

In formative evaluation, the interaction between the evaluator and the explanation also had a significant positive effect on the perception of fairness (β = 0.43, *SE* = 0.20, p < 0.05). When there was no explanation, the student’s perception of fairness brought by the AI algorithm was significantly higher than that brought by the teacher (*M_AI_* = 4.43, *M_Teacher_* = 3.79, *t*(145) = −4.57, *p* < 0.001). However, when there was an explanation, the difference between the perception of fairness under the two evaluators was no longer significant (*M_AI_* = 5.96, *M_Teacher_* = 5,75, *t*(145) = −1.47, *p* > 0.05).

In summative evaluation, the interaction between the evaluator and the explanation still had a significant positive effect on the perception of fairness (*β* = 0.50, *SE* = 0.19, *p* < 0.01). When there was no explanation, the student’s perception of fairness brought by the AI algorithm was significantly higher than that brought by the teacher (*M_AI_* = 4.50, *M_Teacher_* = 3.97, *t*(145) = −4.01, *p* < 0.001). However, when there was an explanation, the difference was no longer significant (*M_AI_* = 5.90, *M_Teacher_* = 5,87, *t*(145) = −1.47, *p* > 0.05).

The results of Study 3 indicated that the presence or absence of an explanation moderated the effect of the evaluator on college students’ perception of fairness, regardless of the type of evaluation. To be more specific, the effect of the evaluator on college students’ perception of fairness was insignificant when an explanation is present and produced a significant difference in the effect of the evaluator on college students’ perception of fairness when no explanation was present.

## Discussion

At the theoretical level, this study has made substantial contributions to the research on the fairness of AI algorithmic evaluation. Firstly, Study 1 findings augment our understanding of how evaluation subjects perceive fairness in the context of digital education. While most existing studies have approached educational evaluation fairness from a social justice perspective or focused on designing pedagogically fair assessments, limited research has examined the influence of evaluation subjects on fairness perception through an organizational justice lens (Thomas and Madison, 2010; McArthur, 2016; Zhu, 2018; Li and Xin, 2019; Roshid et al., 2022). This study extends the application of organizational justice theory to the domain of educational evaluation and sheds light on the informational fairness concerns inherent to diverse evaluators, that is, college students tend to attribute higher perceptions of fairness to AI algorithms than to teachers as evaluators. Secondly, Study 2 results hold significant implications for comprehending the process through which students develop perceptions of fairness in a digital educational environment. This study underscores the mediating role of information transparency. The evaluation of Artificial Intelligence engenders heightened perceptions of fairness due to students’ perception of it as an algorithmic program characterized by greater information transparency. Recent scholarship has increasingly focused on the “black box” problem in AI, highlighting the opacity surrounding AI algorithms and their inner workings (Orlikowski and Scott, 2014; Lee et al., 2015). However, this study offers a contrasting perspective, suggesting that students’ perceptions of the information transparency of AI algorithm evaluations tend to be in the opposite direction and are not or only slightly influenced by their concerns about the algorithms’ transparency. Evidently, under the guidance of AI algorithms, educational evaluation becomes more transparent in students’ views owing to explicit assessment criteria, well-defined processes, and precise outcomes. Lastly, Study 3 findings reaffirm previous research by substantiating the positive impact of explanations on fostering perceptions of fairness, thereby providing valuable guidance for the effective implementation of educational evaluation within the digital education milieu. This study significantly advances our understanding of fairness perception in AI algorithmic evaluation, expanding theoretical frameworks, uncovering informational fairness issues, and offering practical guidelines and the knowledge base for conducting fair educational evaluations in the era of digitization.

Beyond the theoretical implications of this research, further discourse is warranted on potential areas of disagreement within the article. Initially, this investigation applies organizational justice theory to the realm of educational justice, a method not entirely innovative. Numerous preceding studies have employed organizational justice theory to scrutinize equity concerns in education. For instance, Chory-Assad’s (2002) research on classroom justice conceptualized the class as an organization, effectively integrating organizational justice theory into the classroom context. Similarly, we have transposed the concept of performance appraisal into the educational sphere, despite their distinct natures. Performance appraisal of employees constitutes a summative evaluation based on work output, whereas academic performance assessment can be categorized into diagnostic, formative, and summative evaluations (Bloom, 1969, 1987), contingent upon its timing and role in teaching and learning processes. The primary distinction between these two resides in the evaluation type or context. However, they share considerable similarities. Both are evaluative measures of individuals’ achievements, typically quantified. Furthermore, the social relationships underpinning these evaluations are akin. Performance appraisal hinges on superior-subordinate dynamics, paralleling the teacher-student relationship in Chinese academic appraisal. Lastly, both employees and students exhibit significant concern regarding evaluation fairness. It is therefore justified to transpose organizational justice theory to educational justice and recast performance appraisal as academic assessment.

The second salient issue necessitates acknowledging that the evaluation type may influence students’ fairness perceptions. However, we did not consider it a variable but rather a research context. This decision was predicated on several key reasons. Primarily, our focus was on students’ fairness perceptions, not the evaluations themselves. Consequently, we sought to measure students’ fairness perceptions across different contexts to isolate the impact of assessment contexts on fairness perceptions. Our findings suggest that students’ fairness judgments of AI algorithmic evaluations and teacher evaluations exhibit similar trends across all three assessment contexts, indicating that the evaluation type does not significantly influence students’ fairness perceptions. Secondly, we aimed to underscore the subjective nature of assessment contexts rather than differences in assessment types. Despite variations in timing and roles of diagnostic, formative, and summative evaluations, all three essentially constitute subjective assessments. The contrast between human evaluators (teachers) and machine evaluators (AI algorithms) introduces a significant difference in subjective evaluation, likely contributing to variations in students’ fairness perceptions. It is this evaluative discrepancy, rather than the specific assessment context, that accounts for differences in students’ fairness perceptions. Consequently, we opted to use different evaluation types as contexts in our study, rather than as variables.

Furthermore, in the mediator analysis of Study 2, the correlation between “type of evaluator” and “perception of fairness” exhibits a significantly higher magnitude (−0.49) compared to the direct correlation between the two variables (−0.28) in the absence of a mediator variable. To gain a more comprehensive understanding of this unexpected effect, future research should investigate the precise mechanisms by which the mediator variable influences the relationship between “type of rater” and “perception of fairness.” This exploration could involve examining potential mediators such as trust, communication, or perceived competence of the rater. By delving into these variables, we can enhance our comprehension of their contributions to the perception of fairness. In summary, the counterintuitive effect observed in the mediator analysis can be elucidated by considering the mediator variable’s role as either a confounding or intervening variable. Further research is necessary to delve into the specific mechanisms underlying this relationship and identify potential mediators that may help clarify the observed results.

At the practical level, this study provides valuable insights into potential issues in current higher educational evaluation practices. Study 1 reveals that students tend to perceive AI algorithms as fairer when it comes to subjective evaluations. However, it is important to acknowledge that although the integration of new technologies like AI algorithms into the evaluation system is an important trend in educational evaluation reform, AI algorithmic evaluation has yet to achieve widespread adoption, particularly in China. Despite this, Chinese students still express approval of AI algorithmic evaluation, which highlights the significant challenges faced by teachers as the evaluation subjects in terms of fairness. Taking formative assessment as a case in point, it is intended to facilitate direct communication between students and educators. However, in practice, this interaction often resembles a dialog between students and authority figures, rather than a genuine exchange. This relational perception inadvertently hinders students’ active engagement in the assessment and feedback process, subsequently diminishing their perceptions of fairness and trust in teacher evaluations. The findings from Studies 2 and 3 indicate that increasing transparency in information and providing comprehensive explanations of the evaluation process can partially improve students’ perceptions of fairness. It is crucial to recognize that questioning the fairness of teacher evaluation is not a flaw inherent to the evaluators themselves, but rather a problem within the evaluation process. Procedural justice and information justice perspectives suggest that enhancing students’ sense of fairness does not lie solely in replacing the evaluation subjects, but rather in clarifying the roles and responsibilities of different evaluation participants, while simultaneously improving the scientific rigor, transparency, and interpretability of the entire evaluation process. By addressing these aspects, the source of students’ perceived unfairness can be mitigated.

In this regard, several schools have implemented noteworthy initiatives in practice. For instance, a senior high school in Hefei, China, has adopted an artificial intelligence system to capture data generated by students during physical education tests and perform detailed analyses to support teachers in delivering accurate instruction. As an illustration, in the case of long jump assessments, the system assesses whether a student passes or fails while simultaneously capturing screenshots of key movements and recording videos of the complete test. Furthermore, the system provides personalized comments and suggestions for improvement alongside the test results, aiding students in their endeavors to enhance performance. Such comprehensive, transparent, and interpretable evaluation outcomes naturally foster higher perceptions of fairness among students. Furthermore, to enhance the fairness of technology-assisted educational evaluation, it is essential to remain vigilant regarding the potential risks associated with its implementation. On one hand, excessive reliance on technology for evaluation can lead to the phenomenon known as “algorithmic hegemony,” which diminishes the interpretability of evaluation results (Zhang and Qi, 2021). AI algorithms, in particular, involve intricate data collection and processing procedures that are challenging for individuals to comprehend within limited time frames. With a monopoly on technical information, the evaluation results wield an unquestionable authority over individuals, essentially establishing algorithmic hegemony (O’Neil, 2016). For example, in the context of US college admissions, the use of AI algorithms can more accurately predict students’ abilities and facilitate the efficient allocation of financial aid. A case study by Othot (2018) reported that their analysis assisted the New Jersey Institute of Technology (NJIT) in enrolling 173 students while staying within budgetary constraints, resulting in improved recruitment efficiency and cost savings. However, when utilized in educational admissions, these algorithms often operate under the premise of a single theoretical construct. This means they are engineered to focus on one specific aspect or objective of education. In such cases, the algorithm might be programmed to prioritize the “strength of candidate” as its primary metric. This could encompass evaluating factors such as academic accomplishments, standardized test scores, or extracurricular involvement. As a result, the algorithm would rank applicants based on these parameters, potentially offering more admission opportunities to those who excel in these areas. Nonetheless, this singular focus can lead to a narrow interpretation of educational goals. While emphasizing the strength of a candidate, the algorithm might overlook other vital facets of education, such as access and equity. Educational access signifies the opportunity for all individuals, regardless of their background or circumstances, to pursue higher education. Equity, conversely, involves ensuring fairness in treatment, equality of opportunity, and equitable access to information and resources. By concentrating exclusively on the strength of a candidate, the AI algorithm may unintentionally exacerbate disparities in educational access and equity. For example, it might favor applicants from privileged backgrounds who have had more opportunities to develop strong academic or extracurricular profiles, while disadvantaging those from less privileged backgrounds. Therefore, while AI algorithms can streamline the admissions process and identify high-achieving candidates, it’s crucial to consider how their design and implementation might influence broader educational objectives. Future versions of these algorithms should aim to balance multiple educational goals, fostering not only excellence but also access and equity (Liu et al., 2023).

On the other hand, ethical considerations surrounding the use of new technologies must be addressed to ensure the acceptability of the evaluation process. In the case of AI algorithms, evaluations rely on extensive data collection, storage, and analysis, often involving technologies such as facial recognition and body recognition. While these technologies provide robust support for more accurate and scientific evaluations, they also raise concerns about information leakage and privacy infringement (Lu and Gao, 2023). Therefore, before the integration of new technologies into the evaluation system, privacy regulations should be established to safeguard the security and confidentiality of users’ information.

This study extends organizational justice theory to educational evaluation, finding that college students perceive AI algorithms as fairer than teachers. The study highlights the importance of information transparency in shaping students’ perceptions of fairness and contradicts concerns about the “black box” problem in AI. It also confirms that explanations positively impact fairness perceptions. While acknowledging practical challenges in implementing AI in educational evaluation, the study suggests that transparency and comprehensive explanations can enhance fairness perceptions.

## Conclusion

This study contributes to the understanding of fairness perception in AI algorithmic evaluation in the digital context. The findings demonstrate that the evaluator, whether it is an AI algorithm or a teacher, significantly influences college students’ perceptions of fairness in diagnostic, formative, and summative evaluations. Specifically, students perceive AI algorithms as fairer evaluators compared to teachers, with formative evaluation showing the strongest effect. This perception is mediated by the perceived information transparency of AI algorithms, which is perceived to be higher than that of teachers. Additionally, the provision of explanations for the evaluation process moderates the impact of the evaluator on fairness perception. Without explanations, students are more likely to perceive AI evaluations as fairer than teacher evaluations. However, when clear explanations are provided, the influence of the evaluator on fairness perception weakens, leading to no significant difference in perceived fairness between AI algorithms and teachers among college students.

These findings have significant practical implications for educational institutions and policymakers. Recognizing the impact of the evaluator on fairness perception, it is imperative to ensure that both AI algorithms and teachers adhere to the principles of fairness in educational evaluation. This necessitates fostering information transparency in AI algorithmic evaluations and providing comprehensive explanations for the evaluation process. By doing so, educational institutions can enhance students’ perceptions of fairness and promote trust in the evaluation system. From an institutional standpoint, to ensure fair educational assessment, relevant policies, and guidelines should be formulated and implemented. These policies and guidelines should explicitly outline the fairness principles that teachers and AI algorithms must adhere to during the evaluation process. For instance, educational institutions can require teachers to openly disclose grading criteria and evaluation procedures to increase transparency (Carless, 2009; Mhlanga, 2023). Simultaneously, institutions can support teachers utilizing AI algorithms for evaluation by providing technical training and support to ensure they can correctly utilize and explain algorithmic results. Additionally, educational institutions can establish a feedback mechanism allowing students to provide input on the evaluation process, facilitating prompt correction of any potential biases or unfair practices. For policymakers, it is crucial to address fairness concerns in educational assessment and enact corresponding policies and regulations to safeguard student rights. Policymakers can promote standardization and regulation of AI algorithmic assessments, ensuring that the evaluation process possesses verifiability and reproducibility. Furthermore, policymakers should develop measures for privacy protection to prevent personal data disclosure and misuse. In the EU’s Ethics Guidelines for Trustworthy AI, the responsible development of AI is addressed, highlighting its potential to benefit individuals, helping people track their personal data and increase access to education, thus supporting their right to education (European Commission, 2021). Encouraging collaboration between educational institutions and AI algorithm developers, policymakers can work together to establish guidelines and best practices to ensure the fairness and transparency of the evaluation system. These measures will contribute to strengthening students’ perceptions of fairness in educational assessment and building trust in the evaluation system. Moreover, they will provide an effective means to monitor and improve the quality and fairness of educational assessment. By ensuring that the evaluation process aligns with principles of fairness, educational institutions, and policymakers can provide students with a just, objective, and reliable learning environment, fostering their academic achievement and development.

While this study has yielded promising findings, it is important to acknowledge the inherent limitations that may have influenced the results. One limitation arises from the data collection method chosen due to the constraints imposed by the COVID-19 pandemic. The use of an online platform for data collection introduces potential sources of bias, such as sample selection bias and self-selection bias. Additionally, relying on online surveys may introduce technological limitations and digital divide issues, as not all individuals may have had equal access or willingness to participate. Another limitation lies in the scope of variables considered in exploring the relationship between evaluation subjects and perceptions of fairness. Due to feasibility constraints, only two variables were examined in this study. However, it is crucial to recognize that the phenomenon of AI algorithmic evaluation and its impact on perceptions of fairness is multifaceted and complex, warranting further investigation into additional influencing factors.

As we look toward the future, it is important for further research to address the limitations identified in our study and advance our understanding of the topic. One potential avenue for exploration is to expand the sample size to include college students from diverse national and cultural backgrounds. By doing so, researchers can gain valuable insights into the cross-cultural and emotional variations in how AI algorithms and teacher evaluations are perceived in terms of fairness. Including participants from different countries and cultural contexts would allow for a more comprehensive examination of how cultural factors may influence individuals’ perceptions of fairness. It would provide an opportunity to explore whether there are cultural differences in expectations, values, or norms that shape how individuals evaluate the fairness of AI algorithms and teacher assessments. Additionally, considering emotional variations across cultures could shed light on how emotions impact fairness perceptions in educational evaluation settings. For instance, empirical research has demonstrated that students who appraise environmental cues positively (such as the professor’s demeanor and examination settings) are more inclined to conceptualize examinations as opportunities rather than adversities. This positive appraisal extends to their perceptions of other individuals involved in the assessment process (for example, examiners), culminating in an enhanced sense of fairness. Conversely, the presence of negative affective states, such as disappointment or frustration, is associated with a diminished perception of fairness (Ashton-James and Ashkanasy, 2005; Butucescu and Iliescu, 2022). By incorporating a diverse range of participants, future studies can contribute to a more nuanced understanding of the complex interplay between culture, emotions, and fairness perceptions. This expanded scope would enhance the generalizability of findings and help inform the development of more culturally sensitive and equitable educational evaluation practices. Overall, by expanding the sample to encompass college students from diverse national and cultural backgrounds, future research has the potential to deepen our understanding of cross-cultural and emotional variations in the perceived fairness of AI algorithms and teacher evaluations, ultimately contributing to more inclusive and effective educational assessment approaches (Shepherd and Willis-Esqueda, 2018; Ho et al., 2022). Moreover, incorporating more variables into the research design would enable a comprehensive exploration of the mechanisms underlying the effects of AI algorithmic evaluation on fairness perception. These variables could include factors like the transparency and comprehensibility of AI algorithms, the timing and methods of explanations, and individual characteristics like attribution style and trust in technology. In this study, the concept of information transparency in artificial intelligence (AI) algorithms is delineated as the degree to which details pertaining to the evaluation process are disclosed and made accessible to users, drawing on the definitions provided by Florini (2007), Tielenburg (2018), and Wang and Ren (2022). Nevertheless, it is imperative to recognize that AI algorithms are intended to fulfill the objectives of transparency in a context that transcends merely being available and accessible. Various scholars have also highlighted that transparency encompasses the extent to which the internal workings of a system, including the capacity to grasp and articulate the logic underpinning its decisions, are observable and intelligible to human users (Zerilli et al., 2019; De Freitas et al., 2023). Informed by these insights, our subsequent research endeavors will aim to refine the definition of transparency by incorporating additional pertinent dimensions such as explainability and comprehensibility into the AI decision-making framework. This augmented conceptual framework aspires to capture the intricate essence of transparency and provide a more nuanced understanding of the term. Furthermore, future studies can also explore the practical implications of AI algorithmic evaluations and fairness perceptions on students’ academic performance, motivation, and attitudes toward learning. These investigations can shed light on the potential educational implications and inform the development of effective practices and policies in the context of technology-assisted educational evaluation.

This study contributes to the growing body of literature on fairness perception in AI algorithmic evaluation. By examining the impact of the evaluator, information transparency, and explanations, the study provides valuable insights into the factors influencing college students’ perceptions of fairness. These findings have theoretical and practical implications for creating a fairer educational evaluation environment, emphasizing the importance of transparency and explanation provision in promoting fairness of AI algorithmic evaluations. Moreover, this study highlights the need for further research on the long-term effects of AI algorithmic evaluation in the digital education context. Future studies could examine the implications of AI algorithms for student motivation, engagement, and academic performance. Additionally, considering the ethical concerns raised by the use of AI algorithms in educational evaluation, further research should explore strategies to address issues such as algorithmic bias, privacy protection, and accountability. By addressing these issues, we can ensure that educational evaluation remains fair, transparent, and effective in promoting student learning and development in the digital education era.

## Data availability statement

The raw data supporting the conclusions of this article will be made available by the authors, without undue reservation.

## Ethics statement

Written informed consent was obtained from the individual(s) for the publication of any potentially identifiable images or data included in this article.

## Author contributions

FC, JM, JZ, and TH contributed to conception and design of the study. JM organized the database. JM and YW performed the statistical analysis. FC and JM wrote the first draft of the manuscript. YW, JZ, and TH wrote sections of the manuscript. All authors contributed to manuscript revision, read, and approved the submitted version.

## Footnotes

^1^Eta-squared (η^2^) is a measure of effect size for use in ANOVA (Analysis of variance). It is analogous to R^2^ from multiple linear regression. The eta-square effect sizes are interpreted usually as follows: η^2^ = 0.01 indicates a small effect. η^2^ = 0.06 indicates a medium effect. η^2^ = 0.14 indicates a large effect.
